# Rotenone Decreases Hatching Success in Brine Shrimp Embryos by Blocking Development: Implications for Zooplankton Egg Banks

**DOI:** 10.1371/journal.pone.0163231

**Published:** 2016-09-21

**Authors:** Joseph A. Covi, Evan R. Hutchison, Courtney H. Neumeyer, Matthew D. Gunderson

**Affiliations:** 1 Department of Biology and Marine Biology, University of North Carolina at Wilmington, Wilmington, North Carolina, United States of America; 2 Department of Biology, University of Wisconsin-Stevens Point, Stevens Point, Wisconsin, United States of America; University of Connecticut, UNITED STATES

## Abstract

While many zooplankton species recover quickly after the treatment of water resources with the piscicide, rotenone, some fail to reach pretreatment population density or, in rare cases, do not reappear at all. The variable impact of rotenone on zooplankton populations could stem from differences in the capacity of species to switch entirely to anaerobic catabolic pathways in the presence of rotenone, which blocks mitochondrial electron transport. Alternatively, variable responses among species could originate from differences in permeability of dormant life-stages to lipophilic chemicals like rotenone. The purpose of the present study was to determine the effects of rotenone on development, emergence and hatching of zooplankton embryos that lack both the anaerobic capacity to develop in the presence of rotenone and a permeability barrier to prevent the entry of rotenone during dormancy. Post-diapause embryos of the brine shrimp, *Artemia franciscana*, were employed as a model system, because they are permeable to lipophilic compounds when dechorionated and require aerobic conditions to support development. Early development in this species is also well characterized in the literature. Brine shrimp embryos were exposed to rotenone while development was either slowed by chilling or suspended by anoxia. Development, emergence and hatching were then observed in rotenone-free artificial seawater. The data presented demonstrate that rotenone freely diffuses across the embryonic cuticle in a matter of hours, and prevents development and emergence after brief exposures to ecologically relevant concentrations (0.025–0.5 mg L^-1^) of the piscicide. Neither the removal of rotenone from the environment, nor the removal of embryonic water with a hypertonic solution, are sufficient to reverse this block on development and emergence. These data indicate that rotenone could impair recruitment from egg banks for species of zooplankton that lack both an embryonic barrier to the entry of lipophilic compounds and the anaerobic capacity to develop when NADH:ubiquinone oxidoreductase activity is inhibited by rotenone.

## Introduction

Rotenone is a piscicide that is commonly applied in the management of freshwater [1, 2] and coastal marine [3–8] habitats, but the full impact of this important chemical tool on aquatic ecosystems remains unclear after almost 80 years of use [9]. This is especially true for zooplankton communities. It is understood that standard rotenone applications remove the active life-stages of most zooplankton in the water column [10–13]. The copepod, *Paradiaptomus lamellatus* and cladoceran, *Daphnia pulex*, experience 100% mortality after a one hour exposure to 10 μg L^-1^ rotenone [14], which is 4% of the maximum recommended concentration [1]. Active zooplankton populations are presumably restored by recruitment from dormant embryos in the bottom sediments (“egg banks”) [11]. The failure of some species to recover while others rapidly return after rotenone treatment could be explained by variation in susceptibility of the egg bank.

Rotenone is the most biologically active of a class of compounds known as rotenoids, which inhibit NADH:ubiquinone oxidoreductase [15]. By blocking electron transport required for oxidative phosphorylation, rotenoids essentially mimic hypoxia at low concentrations, and anoxia at higher concentrations. This characteristic makes rotenone useful for removing undesirable species of fish that may cause harm to ecosystems, or modifying fish communities for recreational purposes [1]. According to the results of voluntary surveys, over 112,000 kg of rotenone (active ingredient) was used in North America between 1988 and 2002, with a peak use of over 25,000 kg in a single year [2]. Standardized protocols are now in place for treatment of water resources with rotenone [1].

Spatial and temporal variation in rotenone concentrations within a treated water body are difficult to predict. Rotenone concentrations in the water column usually decrease rapidly after application (half-life of 1 d at 23°C to 27°C) [16–18], but half-life of the piscicide in natural lakes can exceed one week [19]. Predicting rotenoid exposure is further complicated by a delayed reappearance of the related rotenoid, rotenolone [16]. The rate of change in rotenone and rotenolone concentration also varies spatially within a single lake [16, 20], perhaps due to variation in distribution and removal. The disappearance of rotenoids in treated water resources is attributed to a complex mix of photolysis, hydrolysis, biotransformation and adsorption to particulates [17–19, 21–24]. In one thorough study, 36% of the rotenone applied to a shallow freshwater pond was detected in the water column one day after treatment with a target concentration of 0.25 mg L^-1^ rotenone [17]. Rotenone levels decreased to 16% of the initial target concentration by day two, and the chemical was undetectable in the water column after one week at water temperatures of 23°C—27°C [17]. At water temperatures of 4°C—5°C, rotenone persisted in the water column for 57 d [17]. In a meticulous study of Diamond Lake (Oregon, USA), 45% of the 0.110 mg L^-1^ rotenone target concentration was still present as rotenone or the active breakdown product, rotenolone, two days after application to waters at 12°C—15°C [16]. Rotenone persisted in the water column of this lake for 32 d, and rotenolone was still present at concentrations near 5 μg L^-1^ when monitoring was terminated after 46 d [16]. Based on these studies, it is reasonable to estimate that zooplankton in lentic waters are exposed to at least 36% of the target rotenone concentration for the duration of the first day following application, and that exposure continues thereafter. Given that it is impossible to mimic the natural dissipation of rotenone and rotenolone under controlled laboratory conditions, any controlled study should, at the very least, test 24 h exposures to rotenone at starting concentrations between 0.04 mg L^-1^ and 0.25 mg L^-1^. This 24 h experimental design is conservative, because exposure to rotenone would continue in field situations as the chemical dissipates over a span of days to weeks.

While it has been reported that rotenone is generally not detected in bottom sediments of treated water bodies [16, 19], it is important to note that these conclusions are largely based on assays of sediment samples that were collected 7 d to 50 d after rotenone application. Unfortunately, the relatively late sampling of sediment in these studies makes it difficult to evaluate the potential impacts of rotenone on benthic organisms. Rotenone possesses a relatively hydrophobic nature (LogP = 4.1) and high sediment partition coefficient [21]. Consequently, it is not surprising that a study of Lake Davis (California, USA) demonstrates the half-life of rotenone and rotenolone to be 3–5 times greater in sediment than in water (average half-life in sediment of 31–32 d) [20]. In a separate study, rotenone concentration in the bottom sediment of a shallow pond increased for 14 d after application to surface waters at 4°C—5°C, and rotenone persisted in the sediment for up to 50 d [17]. These instances of prolonged rotenone presence in bottom sediments suggest that benthic organisms are exposed to the chemical for equal or longer periods than organisms that are exclusively found in the water column. Some support for this is provided by the fact that rotenone persists for one to two months in benthic macroinvertebrates (crayfish and mussels) [17]. Given the potential for prolonged exposure of benthic organisms to rotenone, special attention should be paid to life-stages of invertebrates present in the benthos when rotenone is used. This includes dormant embryos of crustacean zooplankton that make up egg banks required for restoration of populations as part of natural seasonal cycles [25].

Copepods, cladocerans and rotifers reappear in treated lakes within months of rotenone application [10, 16, 20], but taxonomic richness and community composition generally do not return to a pretreatment state for years when long-term monitoring is conducted (reviewed in [9]). The failure of some rotifer and crustacean zooplankton populations to recover could be attributed to differences in predation resulting from altered fish stocks [9]. It is also possible that rotenone blocks recruitment from egg banks for some zooplankton species. In support of this hypothesis, Naess (26) found that resting egg densities for calanoid copepods decreased significantly in a closed marine basin after rotenone treatment, and hatching rates for the remaining eggs were reduced to almost one third of pretreatment levels. Interestingly, this effect was spatially heterogeneous; resting eggs of calanoid copepods at one of three sites within the basin were unaffected while egg density appeared to increase in severity with sediment depth at two other sites [26]. While this study does not provide a statistical evaluation of the effect of depth, it is important to note that the simple loss of older embryos found in deeper sediments indicates that the genetic diversity of the egg bank could be reduced by rotenone treatment. In order to better understand the impacts of rotenone on active zooplankton communities in the water column, it is necessary to assess the impacts of rotenone on dormant life-stages.

Crustacean zooplankton that produce dormant embryos are often adapted to annual or semi-annual removal of active life-stages from the water column by natural succession events [25]. If a normal viable stock of embryos for a single species were to be present in the sediment when rotenone is applied to a lake, then that species should fully recover as soon as the next cycle of environmental cues initiates recruitment from the dormant egg bank. Because recruitment from egg banks of crustacean zooplankton is generally associated with annual cycles in abiotic variables, the populations of most species with a dormant embryonic life-stage should completely recover within a single year following rotenone treatment, provided the egg banks are unaffected. The fact that recovery of copepod and cladocerans populations does not occur in one year (reviewed in [9]) suggests that rotenone negatively impacts recruitment of crustacean zooplankton from egg banks. The present work tests this hypothesis with a readily available and well characterized model organism, *Artemia franciscana*. The data presented demonstrate that rotenone diffuses through the cuticle of a zooplankton embryo and blocks development and emergence in a species that depends on aerobic respiration to support these events.

## Materials Methods

### Chemicals

Artificial seawater (ASW) was made with Instant Ocean^®^ salts (Spectrum Brands, Blacksburg, VA, USA), and salinity was determined with a refractometer. Household bleach was used for dechorionation; all other chemicals were of ACS grade or higher. Rotenone (97% purity) was produced by MP Biomedicals (Santa Ana, CA, USA), and calculations for final concentration corrected for impurity. Rotenone was dissolved in ethanol (EtOH), and final EtOH concentration was 0.01% unless otherwise stated. All aqueous solutions were prepared using ultrapure deionized water with a resistivity of ≥ 18 MΩ cm at room temperature, and ASW was filtered (0.22 μm) before use.

### Animals

Encysted embryos (post-diapause) of the brine shrimp, *A*. *franciscana* (Kellogg) (Great Salt Lake population), were purchased from Artemia International (Fairview, TX, USA) in 2011, and stored at -20°C until use. Embryos were hydrated and dechorionated according to Neumeyer, Gerlach (27) on the day each experiment was started, and all replicates were conducted on separate days. Pre-incubations with rotenone were conducted in glass flasks. Hatching tests were conducted in sterile polystyrene 12-well culture plates or autoclaved glass Erlenmeyer flasks. When hatching tests were conducted in Erlenmeyer flasks, subsamples were transferred by pipette to petri dishes for stage identification. Development, emergence and hatching were assessed and recorded according to Neumeyer, Gerlach (27). In brief, individuals were first categorized by classic emergence and hatching terms: encysted embryo, first stage of emergence (E1), second stage of emergence (E2) and swimming larva (reviewed in [27]). Development of the embryo is not always synchronized with emergence. Consequently, a second term was added to each of these classic categories to describe the state of embryo/naupliar development visible through the transparent embryonic cuticle that remains after dechorionation. In brief, early development (ED) is defined as an embryo for which bilateral symmetry and segmentation are not visible under a dissecting light microscope with 50-100X magnification [27]. Intermediate development (ID) is defined as an individual with head segmentation, antennal buds and muscle twitching visible under a dissecting light microscope, and late development (LD) is characterized by the presence of an elongated thoracoabdomen and well developed antennal setae with the possibility of coordinated muscle contractions for antennal movement [27]. Combining these developmental terms with the classic emergence and hatching terms (e.g. E1-ED for early development in the first stage of emergence) provides a developmental category that remains valid under conditions that disrupt the coordination of developmental events with emergence and hatching.

### Temperature

An incubation temperature of ~0°C was achieved by placing flasks in covered foam-insulated containers filled with crushed ice and stored in a refrigerator. Incubations at 4°C were monitored at the start and finish of treatments with a standard research-grade mercury thermometer (-20°C to 110°C range) immersed in a mock control flask. For bench-top incubations, room temperature is reported as 22°C based on thermostat setting checked against a standard research-grade mercury thermometer. When an environmental chamber was used to control light and temperature, temperatures are reported with a range recorded using a HOBO U12 data logger (Onset Computer Corporation, Bourne, MA, USA).

### Time-dependent effects of rotenone exposure

Dechorionated embryos (0.6 g) were preincubated under constant darkness for 0 h, 1 h or 24 h in 200 ml 0.25 M NaCl containing 0.5 μg ml^-1^ rotenone. Preincubations were carried out at ~0°C using pre-chilled solutions. Embryos were washed by resuspending in fresh 0.25 M NaCl three times at room temperature to remove rotenone from the medium. Embryos were then decanted into a DuraWipe^®^ cloth (Chicopee Inc., Charlotte, NC, USA), blotted dry, and 0.3 g were transferred to a clean 200 ml Erlenmeyer flask containing 25 ml of 25‰ ASW for hatching tests. Embryos were hatched at room temperature under irregular room lighting with orbital shaking at 125 rpm. Flask tops were covered with a dry DuraWipe^®^ cloth to limit evaporation. Hatching success was assessed by sampling culture 43 h after start of shaking; a mean±s.e.m. of 349±28 individuals were assessed for each treatment group. Salinity increased by 3.3‰ per 22 h period in a single test flask.

### Concentration-dependent effects of rotenone

The concentration-dependent effects of rotenone were assessed using two methods based on techniques described previously [27]. The protocol for concentration-dependent experiment No. 1 was identical to the method described above for testing time-dependent effects of rotenone, except for the following. First, the mass of embryos in the pretreatment was decreased to 0.15 g to speed up processing. Second, the embryos were preincubated for 24 h in 0.125, 0.25 or 2.5 μg ml^-1^ rotenone; the control excluded rotenone. Third, hatching success was assessed by sampling of room temperature culture after 64–67 h; a mean±s.e.m. of 185±12 individuals were assessed for each treatment group.

Concentration-dependent experiment No. 2 employed an improved technique for culturing that reduced light exposure, decreased evaporative water loss, eliminated physical agitation, and allowed visual inspection of the same embryos over a 68 h period [27]. In brief, dechorionated embryos (0.15 g) were preincubated on crushed ice under constant darkness for 24 h in 100 ml 0.25 M NaCl containing 0.025, 0.05, 0.1, 0.25 or 0.5 μg ml^-1^ rotenone. A preincubation without rotenone (0 μg ml^-1^) was included as a control. The final EtOH concentration was 0.025% for all treatments. Embryos were washed by resuspension twice in 0.25 M NaCl, and once in 25‰, ASW before transferring to 12-well plates with plastic lids for hatching studies; for each treatment type, 30–33 embryos were placed in each of 4 wells, providing a mean±s.e.m. of 123±2 individuals for a single treatment group. Plates were maintained at 22±0.5°C under constant darkness in an LI20P incubator (Sheldon Manufacturing Inc., Cornelius, OR, USA) to limit photo-degradation of rotenone, and development was monitored periodically over 68 h. Salinity increases by less than 2% over 72 h under these conditions [27].

### Effect of washing on rotenone sensitivity

Dechorionated embryos (0.6 g) were preincubated for 24 h under constant darkness in 200 ml of 0.25 M NaCl on crushed ice with or without 0.5 μg ml^-1^ rotenone. Embryos were then washed by resuspending three times in 0.25 M NaCl at ~0°C, decanted into DuraWipe^®^ cloth, and blotted dry. Embryos (0.5 g) were transferred to a flask containing 100 ml of 26% NaCl, and stored at 4°C under constant darkness for 24 h. The 26% NaCl treatment was used to remove the bulk of embryonic water by osmosis. Embryos were washed two more times by resuspension in 0.25 M NaCl at ~0°C, decanted into a DuraWipe^®^ cloth and blotted dry. For rehydration, blotted dry embryos (0.15 g) were immediately placed in 25 ml of 25‰ ASW at ~0°C. After 24 h, the embryos were transferred to flasks containing 25‰ ASW at 22°C, and 43 h hatching tests were conducted as described above for time-dependent tests.

### Effect of anoxia-induced quiescence on rotenone sensitivity

To test the effect of anoxia-induced quiescence on rotenone sensitivity, 0.15 g of dechorionated embryos were preincubated under constant darkness for 5 d at 4°C in air-tight canning jars (Ball Corp., Broomfield, CO, USA) containing 100 ml of 0.25 M NaCl with or without 0.125 μg ml^-1^ rotenone. To test the effect of culture density on rotenone sensitivity, a third treatment with 0.125 μg ml^-1^ rotenone was included that differed only by the addition of 0.9 g of embryos instead of 0.15 g. Before adding rotenone or embryos, the incubation solutions were bubbled with nitrogen (N_2_) gas for 30 min to displace environmental oxygen (O_2_). Bubbling with N_2_ continued during the addition of rotenone and embryos. At the end of the 5 d exposure, embryos were washed by resuspending three times in 25‰ ASW before transferring to 12-well plates; for each treatment type, 22–85 embryos were placed in each of 4 wells containing 1 ml of 25‰ ASW. This provided a mean±s.e.m. of 198±10 individuals for each treatment group. Plates were covered with a damp DuraWipe^®^ cloth cover that draped over into a water bath under each plate. Hatching success was assessed after 68 h at room temperature under a 12:12 L:D cycle; an incubator was not available for this experiment.

To determine whether or not rotenone was still present in the incubation media after the 5 d anoxic incubations, media from all anoxic incubations was collected as embryos were removed by filtration. Freshly dechorionated embryos (0.15 g) were then added to this media, and stored at 4°C under constant darkness. After 24 h, embryos were washed by resuspending three times in fresh 25‰ ASW before transferring to 12-well plates; for each treatment type, 29–126 embryos were placed in each of 4 wells containing 1 ml of 25‰ ASW. This provided a mean±s.e.m. of 241±11 individuals for each treatment group. Hatching tests were conducted in the same manner as for embryos used in the initial anoxic experiments.

The effects of temperature on rotenone sensitivity when exposure occurred during anoxia-induced quiescence were also tested. Conditions were identical to the initial test of rotenone exposure during anoxia-induced quiescence, except that separate incubations at 4°C and 22°C were included. For each treatment, 4–85 embryos were placed in each of 12 wells containing 1 ml of 25‰ ASW. This provided a mean±s.e.m. of 247±30 individuals for each treatment group.

### Data analysis

Differences between two means were assessed by Student’s t-test. Differences among more than two means were assessed by one-way ANOVA followed by Tukey’s post-hoc multiple comparison or two-way ANOVA followed by Bonferroni’s post-hoc multiple comparison, α = 0.05. When periodic observations of the same individuals were made, the peak in abundance of a life-stage was identified as the mean value that was: 1) not significantly less than any other time point, 2) significantly greater than at least one non-zero mean for an earlier time point and 3) significantly greater than at least one non-zero mean for a later time point. If the mean abundance for two or more times points satisfied these criteria, then the peak was assumed to span these time points. Student’s t-test and one-way ANOVA were conducted with JMP 11.0 (SAS Institute, Cary, NC, USA). Two-way ANOVA and Bonferroni multiple comparison were conducted with SigmaPlot 12.5 (Systat Software, San Jose, CA, USA). All analyzed data are available in a single supporting information file (S1 Fig).

## Results

### Time-dependent effects of rotenone

**Fig 1:**
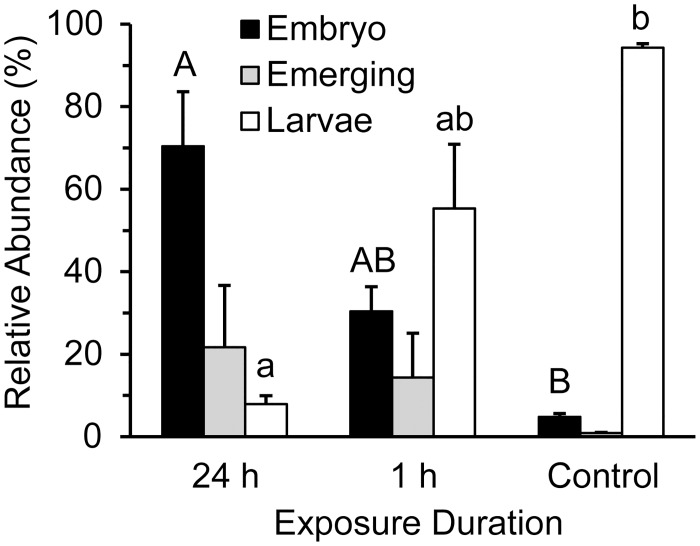
Time-dependent effects of pre-exposure to rotenone. Emergence and hatching of *A*. *franciscana* were assessed by endpoint assay after 43 h of development in rotenone-free ASW. Duration of pre-exposure to 0.5 μg ml^-1^ rotenone during preceding 24 h was varied. Relative abundance of three developmental stages plotted as mean±s.e.m. with percent values calculated from evaluation of 349±28 embryos per treatment replicate, n = 3; ANOVA and Tukey’s post-hoc test used to compare three treatment types for each developmental stage; shared letters indicate no significant difference among means with upper case used for encysted embryos and lower case for larvae. No significant differences identified for emerging embryos.

Emergence and hatching in *A*. *franciscana* are negatively impacted in a time-dependent manner when dechorionated embryos are preincubated with rotenone and then hatched in rotenone-free ASW. Slightly over 90% of embryos in control treatments hatched into swimming larvae within 43 h when hatching occurred in Erlenmeyer flasks containing 25‰ ASW aerated by orbital shaking under fluorescent room lighting with a culture density of 1.5 mg embryo per ml ASW (Fig 1). When embryos were preincubated on crushed ice for 1 h under aerobic conditions with 0.5 μg ml^-1^ rotenone prior to hatching tests, 57% hatched successfully and 13% partially emerged, but these values were not significantly different from the control (Fig 1). When embryos were preincubated on crushed ice for 24 h under aerobic conditions with 0.5 μg ml^-1^ rotenone prior to hatching tests, the number of embryos that failed to initiate emergence increased 16 fold relative to the control (Fig 1; p = 0.0013) and the number of individuals that hatched as larvae decreased by 86±1% relative to the control (Fig 1; p = 0.0036).

### Concentration-dependent effects of rotenone

**Fig 2:**
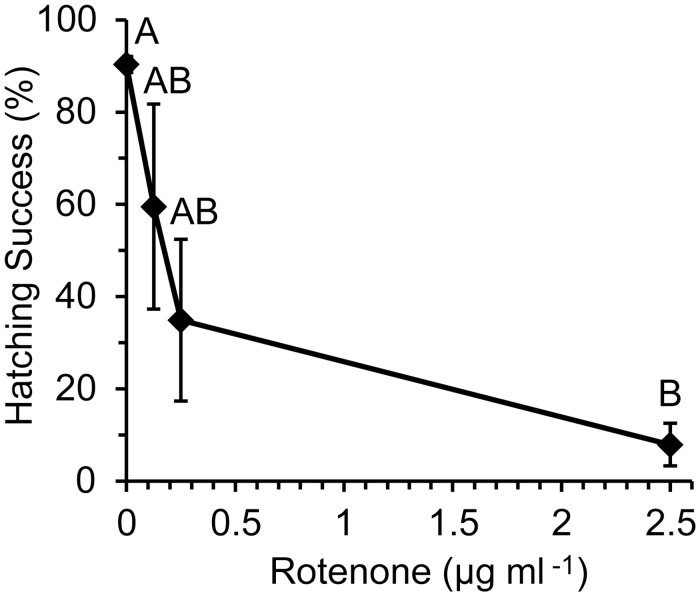
Concentration-dependent effect of pre-exposure with rotenone (method No. 1). Hatching success for *A*. *franciscana* was assessed by end-point assay after 64–67 h in rotenone-free ASW under room lighting with aeration by orbital shaking. Concentration of rotenone during 24 h preincubation at 0°C was varied. Relative abundance of larvae plotted as mean±s.e.m.; n = 3 with 185±12 embryos per treatment replicate; ANOVA and Tukey’s post-hoc test used to compare means; shared letters indicate no significant difference.

**Fig 3:**
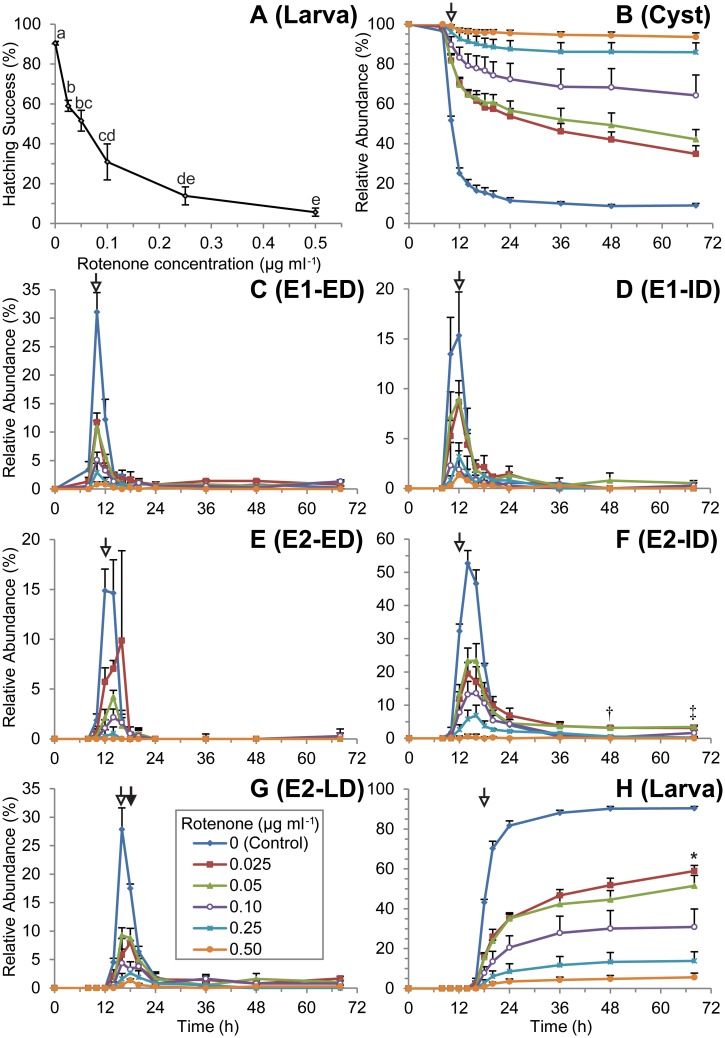
Concentration-dependent effect of pre-exposure with rotenone (method No. 2). Early development, emergence and hatching in *A*. *franciscana* were assessed by periodic observation over 68 h in static 12-well plates containing rotenone-free ASW, and maintained under constant darkness. Concentration of rotenone during 24 h preincubation at 0°C was varied. Hatching success was assessed after 68 h at 22°C (A). Development, emergence and hatching were also assessed at 2 h intervals for encysted embryonic (B), emergence 1 (C,D), emergence 2 (E-G) or larval (H) stages according to nomenclature of Neumeyer, Gerlach (27). Data plotted as mean±s.e.m.; n = 3 with 123±2 embryos per treatment replicate; ANOVA followed by Tukey’s post-hoc test used to compare means for treatment types at each time point; shared letters indicate no significant difference; open arrows identify time-point when 0.025 μg ml^-1^ rotenone treatment first significantly altered abundance relative to the control; †, mean values for 0.025 μg ml^-1^ and 0.05 μg ml^-1^ treatments significantly greater than all other treatments; ‡, mean for 0.025 μg ml^-1^ significantly greater than control treatment while 0.05μg ml^-1^ treatment is significantly greater than all other treatments at the same time point; *, mean value at 68 h significantly different from 36 h as determined by two-tailed Student’s t-test; solid arrow indicates statistically supported peak shift for rotenone treatments, relative to the control.

**Fig 4:**
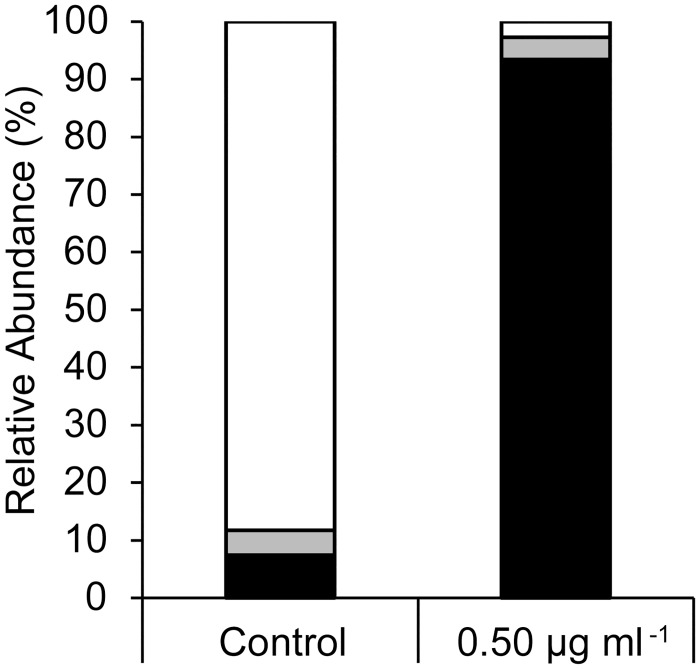
Washing embryos, and removing bulk embryonic water by a cycle of dehydration and rehydration, fails to reverse the effects of rotenone pre-exposure on emergence and hatching. Relative abundance of three developmental stages at 43 h is displayed: black for embryo, grey for emergence stages, and white for larvae. Control lacked rotenone; percent values calculated from evaluation of 162 or 183 individuals for control and rotenone treatments, respectively.

Hatching of *A*. *franciscana* decreased in a concentration-dependent manner when dechorionated embryos were preincubated for 24 h with rotenone, and then hatched in rotenone-free ASW (Figs 2 and 3). Less than 10% of embryos hatched on average if they were preincubated for 24 h in 0.5 μg ml^-1^ or 2.5 μg ml^-1^ rotenone (Figs 1–3). This effect was not reversed by rigorous washing that included the removal of embryonic water by a cycle of dehydration in a hypersaline solution followed by rehydration in rotenone-free ASW (Fig 4).

Subtle impacts of both rotenone, and the method of incubation, became apparent when embryos were monitored every two hours during hatching (Fig 3). Rotenone delayed the initiation of emergence in a concentration dependent manner, as assessed by timing for the disappearance of the embryonic (cyst) stage (Fig 3B). Rotenone also decreased the total number of individuals that reached later stages of development, and in a concentration-dependent manner (Fig 3C–3H). Variability in the susceptibility of hatching success to rotenone decreased when embryos were hatched in static 12-well plates maintained at 22±0.5°C under constant darkness in an incubator (method No. 2) (Fig 3) instead of Erlenmeyer flasks aerated by orbital shaking under room lighting (method No.1) (Figs 1 and 2). The impacts of low rotenone concentrations were also more pronounced in the static plate incubations of method No. 2 (c.f. Figs 2 and 3A).

A one-way ANOVA and Tukey’s post-hoc multiple comparison test were applied to determine the time at which the abundance of a specific developmental stage peaks within each treatment type (see data analysis methods). To simplify presentation, mean comparisons are not indicated with traditional letters (Fig 3). Rotenone did not significantly shift the time at which abundance of the E1-ED, E1-ID, E2-ED, or E2-ID life-stages, as defined by Neumeyer, Gerlach (27), peaked for any individual treatment type (Fig 3C–3F). However, all concentrations of rotenone tested did delay the peak in abundance of the E2-LD stage relative to the control (Fig 3G).

For control embryos, and embryos preincubated in rotenone concentrations exceeding 0.025 μg ml^-1^, hatching rates at 36 h were statistically indistinguishable from that at 48 h or 68 h, but embryos preincubated with 0.025 μg ml^-1^ continued to hatch between 36 h and 68 h (Fig 3H; p<0.05). A similar qualitative increase in hatching was apparent for embryos preincubated in 0.05 μg ml^-1^ rotenone (Fig 3H). A small percentage of embryos (<5%) preincubated in the lowest concentrations of rotenone (0.025 μg ml^-1^ or 0.05 μg ml^-1^) arrested in the E2-ID stage (Fig 3F). Arrested development was not observed for any other rotenone concentration or developmental stage (Fig 3F).

### Effect of anoxia-induced quiescence on rotenone sensitivity

**Fig 5:**
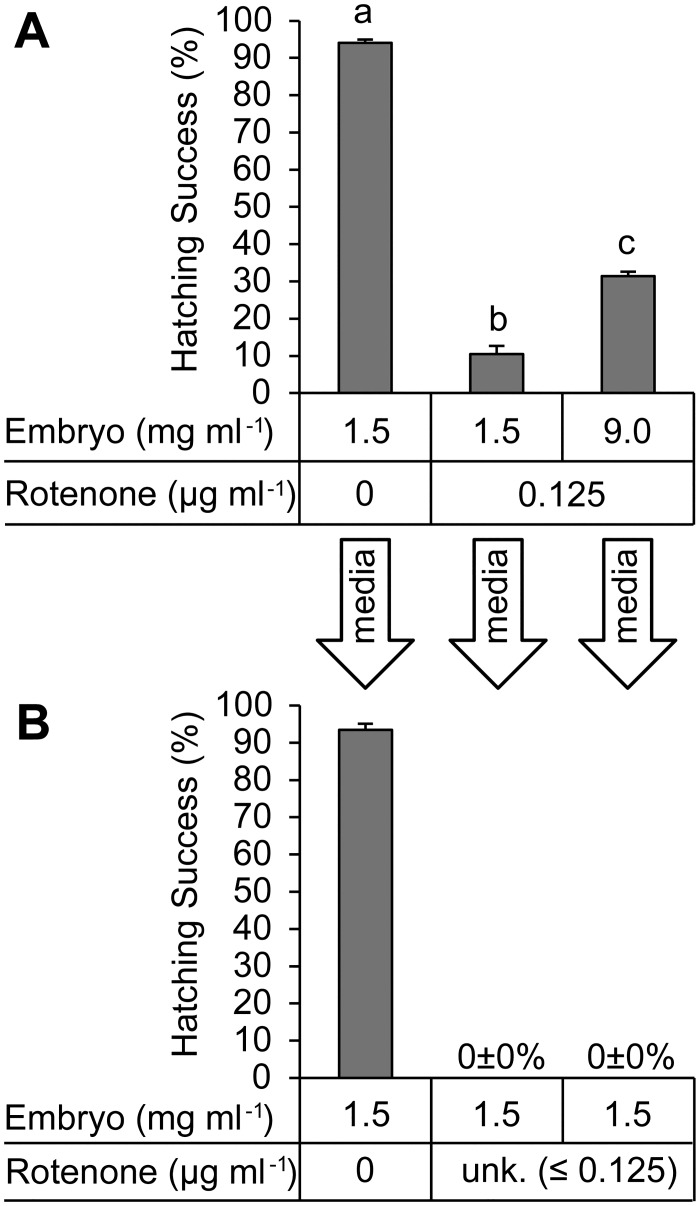
Both anoxia-induced quiescence and increased embryo abundance partially protect against rotenone during pre-treatment. A) Embryos were pre-incubated in the presence or absence of rotenone at two different embryo densities for 5 d under anoxic conditions, after which they were washed and hatch-tested over a subsequent 68 h in rotenone-free ASW. This method tested the effect of embryo abundance on susceptibility to rotenone during prolonged exposure, which could occur during a natural bout anoxia-induced dormancy. B) It was hypothesized that toxicity of the media would decrease in direct proportion with embryo abundance. To test this, residual toxicity of the incubation medium from the 5 d anoxic pre-incubation was subsequently tested in a 24 h aerobic pre-exposure at 4°C with a fresh preparation of dechorionated embryos. Hatching success was again assessed after subsequent washing and 68 h incubation in rotenone-free ASW. Arrows indicate the origin of media from experiments in panel A that were used in the follow-up test in panel B. Embryo abundance and rotenone concentration for each of the two experiments are listed under each graph. Residual rotenone concentrations were not experimentally determined, and are therefore listed as unknown (unk) in the follow-up media test. Data plotted as mean±s.e.m.; n = 3 with 198±10 or 241±11 individuals per replicate treatment group for the initial anoxic experiment or the subsequent media toxicity tests, respectively; one-way ANOVA followed by Tukey’s post-hoc test used to compare three treatment types for each developmental stage; different letters indicate significant differences between means; statistical tests not used when zero hatching was observed for treatments.

Hatching success in rotenone-free ASW under normoxic conditions decreased when embryos were pre-exposed to rotenone during quiescence induced by bubbling of media with N_2_ gas to remove oxygen from the ASW (Fig 5A). Hatching of embryos preincubated under anoxic conditions for 5 d at 4°C in the presence of rotenone (0.125 μg ml^-1^) was significantly less than that for the rotenone-free control (Fig 5A). Increasing the ratio of embryos to ASW during the preincubation with rotenone mitigated the effect of rotenone on hatching; nearly threefold more embryos hatched in the treatment group with an embryo density of 9 mg ml^-1^ than in the treatment group with 1.5 mg embryo ml^-1^ (Fig 5A). A subsequent test of the media from this experiment under aerobic conditions demonstrated that this difference was not due to an insufficient concentration of rotenone (Fig 5B).

Preincubation with 0.125 μg ml^-1^ rotenone at 4°C under anoxia for 5 d decreased hatching by 90% (Fig 5A), which is greater than that predicted by 24 h pre-exposure at ~0°C (Fig 3). Increasing the ratio of embryos to ASW decreased the impact of rotenone on hatching success (Fig 5A). The block of hatching by rotenone was more pronounced in a 24 h normoxic preincubation at 4°C than the 5 d anoxic preincubation at the same temperature (Fig 5). A normoxic preincubation with rotenone for 24 h at 4°C also decreased subsequent hatching in rotenone-free ASW to a greater extent than a 24 h preincubation at ~0°C (c.f. Figs 3 and 5B). Hatching success of control embryos preincubated for 5 d under anoxic conditions was nearly identical to that for control embryos preincubated for 24 h when temperature was maintained at 4°C or ~0°C (c.f. Figs 3, 5A and 5B).

**Fig 6:**
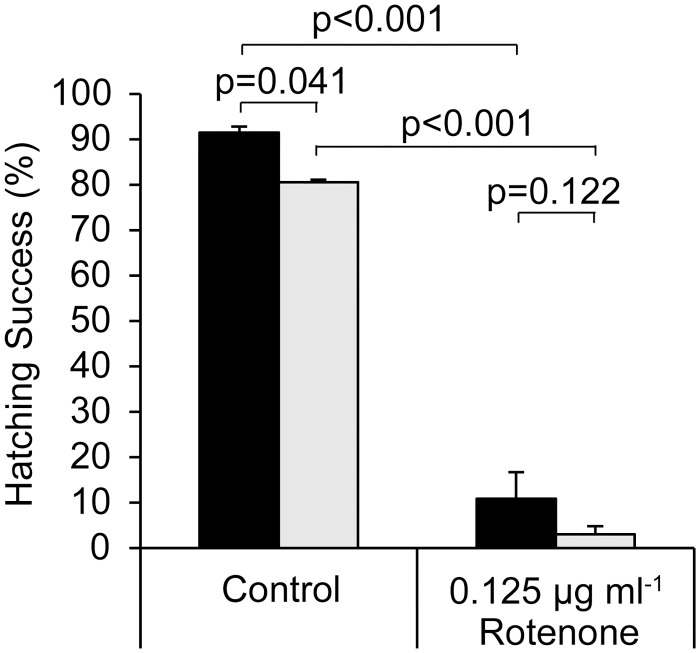
Increasing temperature does not increase the sensitivity of hatching to pre-incubation with rotenone for 5 d under anoxia. Hatching success was assessed in rotenone-free ASW under normoxic conditions at 22°C after anoxic pre-incubation for 5 d at 22°C (grey bars) or 4°C (black bars) in the presence or absence of rotenone. Data plotted as mean±s.e.m.; percent hatch calculated from evaluation of 247±30 embryos per individual replicate, n = 3; p-values for Bonferroni multiple comparison test are displayed.

A two-way ANOVA was employed to determine the effects of anoxic preincubation temperature on hatching success and sensitivity to rotenone (Fig 6). The effects of anoxic preincubation temperature, *F*_1, 8_ = 8.650, *p* = 0.019, and rotenone, *F*_1,8_ = 619.346, *p* < 0.001, on subsequent hatching success were statistically significant. However, based on a Bonferroni multiple comparison test, the small decrease in hatching success that occurred when the anoxic preincubation temperature was increased from 4°C to 22°C was only statistically supported for the control (rotenone-free) treatment (Fig 6). There was no significant interaction between temperature and rotenone exposure during anoxic preincubation, *F*_1,8_ = 0.246, *p* = 0.633.

## Discussion

The objective of the present study was to determine the effects of rotenone on development, emergence and hatching in crustacean zooplankton when temporary exposure occurs during a preceding bout of embryonic dormancy. Unfortunately, embryos of crustacean zooplankton known to inhabit lakes where rotenone is commonly used are not readily available in the quantity or quality required of a toxicology model. Post-diapause embryos of the brine shrimp, *A*. *franciscana*, were used as a model system because early development is well characterized in this readily available species. The data presented demonstrate that 1) rotenone freely diffuses across the embryonic cuticle in a matter of hours, 2) rotenone prevents development and emergence in dechorionated embryos at ecologically relevant concentrations and 3) removal of rotenone from the environment is not sufficient to reverse its blockade of development and emergence. We argue that rotenone could impair recruitment from egg banks for species that lack a permeability barrier to lipophilic compounds, and the anaerobic capacity to develop in the presence of this piscicide.

Many species of crustacean zooplankton that are found in freshwater, hypersaline or coastal marine waters, including representatives of the Anostraca, Copepoda and Cladocera, produce embryos capable of remaining dormant for decades to centuries [25, 28–33]. In order to survive for such long periods, these embryos must enter a nearly ametabolic state, as has been demonstrated for *A*. *franciscana* [34–36]. In the absence of metabolic activity for homeostatic processes, the cyst wall that surrounds an embryo would need to block the movement of ions and hydrophilic compounds in order to prevent loss by diffusion. The fact that embryos of many cladocerans, ostracods and copepods survive transit through vertebrate digestive systems [37] suggests that they are at least impermeable to protons. By contrast, several studies indicate that the protective barriers of copepod (*Eurytemora afinis*, *Centropages hamatus*, *Acartia* spp. and *Tortanus forcipatus*) and cladoceran (*Daphnia magna*) embryos are permeable to organic pollutants [26, 38, 39]. In *A*. *franciscana*, a thick proteinaceous chorion of maternal origin provides a barrier to lipophilic compounds [40–42]. The underlying embryonic cuticle provides a barrier to protons, metals, simple salts and hydrophilic compounds [43–45]. When the chorion is removed, embryos of *A*. *franciscana* resemble the unmodified diapause embryos of copepods [27], and development is unaltered [27, 46]. The present work demonstrates for the first time that these dechorionated embryos are permeable to the lipophilic chemical, rotenone (Figs 1–6), and that rotenone decreases hatching success in *A*. *franciscana* in a manner consistent with field tests on the embryos of copepods, including those of *E*. *afinis*, *C*. *hamatus* and *Acartia clausi* [26].

The results of the present study could explain variability in recovery times for zooplankton populations following rotenone treatment. Early studies noted that cladocerans and copepods were rapidly removed from the water column after rotenone application, while rotifer abundance and diversity were only moderately reduced [12, 13]. In one example of ponds treated with 15 ppb rotenone, active zooplankton communities recovered within 8 months, although relative abundance of species within the cladoceran community was not restored to levels predicted by communities in control ponds [10]. In a study of two mountain lakes in Alberta, Canada, only 40% of all crustacean zooplankton species recovered 9 months after treatment of the lakes in September with 37.5 ppb rotenone [11]. Rotifers were more resilient; populations of two species of rotifer in the lakes were unaffected (*Kellicottia longispina* and *Keratella cochlearis*) and the remainder recovered the following summer [11]. In wetlands treated with 150 ppb rotenone, the abundance of calanoid copepods only reached 17% of pretreatment abundance a full year after treatment, while populations of *Ceriodaphnia*, *Daphnia* and cyclopoid copepods recovered in less than one year [47]. Together, these data demonstrate that recovery of zooplankton from rotenone treatment is both taxon and concentration-dependent. The failure of some species to recover after a full year could be explained by lack of recruitment from the egg bank. The present study provides some support for this hypothesis by demonstrating that hatching success in a model species decreases steadily as the concentration of rotenone is increased across an ecologically relevant range (25 ppb to 250 ppb) for an ecologically relevant duration of exposure (1–5 d).

Sensitivity to rotenone was greater when embryos developed in static plates protected from light than under room lighting with aeration by orbital shaking (c.f. Figs 2 and 3A). This is clearly not due to an effect of light on hatching, because 88%–96% of control embryos hatch within 72 h regardless of whether they develop under irregular room-lighting (Figs 1, 2 and 4), a 12:12 L:D cycle (Figs 5 and 6) or constant darkness (Fig 3). It is possible that the increased sensitivity observed in embryos incubated in static plates protected from light results from an increase in rotenone half-life. No attempt was made in the present study to mimic light exposure in a natural environment, because light intensity and duration will vary with time of year, latitude, cloud cover, water depth, water clarity, vegetation, sediment depth, sediment composition and physical perturbation of the water and sediment. Likewise, no attempt was made to mimic the impact of a natural sediment matrix on stability or bioavailability, because the sediment matrix will vary among water resources where rotenone is used. Instead, the present study assessed the impact of a range of concentrations (Fig 3) that occur in treated water resources within the first 48 h of rotenone application [16, 17, 20]. Given that light exposure, physical agitation and oxygen tension are expected to be low for embryos in bottom sediments, static incubation in the absence of light, as was employed for the experiments described in Fig 3, is perhaps the most ecologically relevant condition for a controlled study that does not test exposure in a specific sediment matrix.

The suppression of development and emergence by rotenone (Figs 2 and 3) is moderately attenuated by anoxic conditions (Fig 5), an increase in embryo culture density (Fig 5A) and a decrease in temperature (Fig 6). The first of these might be easily explained; anoxia induces a state of developmental and metabolic suspension referred to as quiescence [31, 35]. Lack of metabolic activity during exposure to rotenone most likely allows embryos to take up enough rotenone to establish a state of chemical anoxia before normoxic conditions are restored. This is supported by the fact that non-developing embryos in rotenone treatments generally do not decay over 68 h of monitoring (data not shown). It is plausible that these embryos are in a state of chemically-induced quiescence.

The effects of temperature and culture density on hatching are most likely independent of rotenone. The protection offered by increasing culture density (Fig 5A) aligns with previous work showing that emergence and hatching are density-dependent [27]. The protection offered by lowering temperature is most likely unrelated to the diffusible factor of embryonic origin described by Clegg [48], because temperature and culture density were relatively low in the present study. Regardless of the origin, these effects necessitate either the use of a range of culture densities and temperatures, or standardization of both, for future work.

It may be unreasonable to expect that zooplankton communities will, or even should, return to pretreatment states following the dissipation of rotenoids from a system when predatory fish stock are being intentionally altered. That said, it is important to protect dormant life-stages of all zooplankton species, because they have clear ecological and evolutionary roles [25, 49]. While we do not presume to have an answer, it is important to ask if the potential loss of decades to centuries of genetic diversity within an egg bank is something that should be considered “reasonable”, as per the Standard Operating Procedure Manual for rotenone use [1]. Benthic-pelagic coupling is too often ignored [49]. In the case of rotenone use in water resources, it may even be counterproductive not to consider it more carefully, because speeding the recovery of non-target zooplankton could enhance the recovery of more prominent desired species [10]. Unfortunately, a lack of common methods for treatment and monitoring make it impossible to make definitive statements about the effects of rotenone on invertebrate populations [9]. We propose that zooplankton models like the one used in the present work would provide a consistent test of toxicity for species that are susceptible in the dormant embryonic state. For initial toxicological assessments, dechorionated embryos could even be combined with natural sediments to account for interactions between rotenone and the sediment matrix. The embryos of *A*. *franciscana* are excellent substitutes for freshwater species, because embryo development is unaltered by incubation in freshwater prior to emergence [50]. Exposure to low salinity during early development actually improves hatching success [27] and subsequent growth [51] in *A*. *franciscana*, when compared to culturing in normal seawater.

## Supporting Information

S1 Fig Supporting Information Data for Figs 1–6. Data used to produce Figs 1**–**6 are summarized on separate tabs in a single spreadsheet document.
(XLSX)
